# News and Coming Events

**Published:** 1907-06-08

**Authors:** 


### NEWS AND COMING EVENTS.

It is proposed to re-establish a children's ward in Addenbrooke's Hospital, Cambridge.

In response to the appeal for £18,000 made by the committee of the Bristol General Hospital, a sum of £13,000 has already been subscribed.

A ball will be given at the Grafton Galleries on Waterloo Day, June 18, under the patronage of Princess Louise, Duchess of Argyll, in aid of the funds of the Royal Waterloo Hospital.

In 1902 Lord Mount Stephen, with a view to increasing the endowment of the Royal Infirmary at Aberdeen, presented it with securities yielding an annual income of £1,000. He has now given a further donation of £10,000 towards the funds of the institution.

The new wing and operating-theatre at the West Cornwall Miners' and Women's Hospital, Redruth, has been opened. The new building stands between the two wards of the old hospital, and is approached from both by a broad corridor. The walls and ceilings are of adamant cement, finished with parapan paint. The theatre is fitted with the most modern appliances, and is in every way suited to its purpose.

The degree of Doctor of Medicine has been conferred upon Dr. Wilfred T. Grenfell, C.M.G., by the University of Oxford, *honoris causa*, at a convocation on May 28, in recognition of the splendid work he has achieved in improving the social condition of the Labrador fisherfolk. The honour bestowed upon him by his old University is all the more marked by reason of its being the first honorary M.D. degree conferred at Oxford.

Medical men who have had the privilege of studying in Paris will be interested to know that the English pupils of the late M. Alexandre Beljame, for many years Professor of English at the Sorbonne, intend to raise a fund to perpetuate his memory by the erection of a marble tablet in his lecture-room. The professor was well known to English medical students, to whom he endeared himself by his courtesy and kindliness. The secretaries of the fund are M. Clermont, of the Lycée de Sailly, and Mlle. Scott, of the Lycée Molière.

London School of Clinical Medicine ("Dreadnought" Hospital, Greenwich).—The following is the programme for the summer session: Monday, Medicine, Sir Dyce Duckworth, 2.15 p.m.; Surgery, Mr. William Turner, 3.15 p.m.; Throat and Ear, Dr. St. Clair Thomson, 4 p.m. Tuesday, Medicine, Dr. R. Tanner Hewlett, 2.15 p.m.; Surgery, Mr. Carless, 2.15 p.m.; Diseases of the Skin, Mr. Malcolm Morris, 4 p.m. Wednesday, Medicine, Dr. Frederick Taylor, 2.15 p.m.; Ophthalmology, Mr. Cargill, 3.30 p.m. Thursday Medicine, Dr. Guthrie Rankin, 2.30 p.m.; Surgery, Sir William Bennett, 3.15 p.m.; Radiography, Mr. Mackenzie Davidson, 4 p.m. Friday, Medicine, Dr. Rose Bradford, 2.15 p.m.; Surgery, Mr. McGavin, 3.15 p.m. Operations each day at 2.30. Out-patient Demonstrations: Surgical, daily, 10 a.m.; Medical, daily, 10 a.m.; Ears and Throats, Mondays and Thursdays, 12 a.m.; Eyes, Wednesdays and Saturdays, 11 a.m.; Skins, Tuesdays and Fridays, 12 a.m. Special Lectures: June 10, (Monday), Mr. Wm. Turner, "Empyema"; Wednesday, June 12, Dr. F. Taylor, "Diagnosis of Typhoid Fever."

The Wagga-Wagga Hospital, N.S.W., has started a rebuilding scheme which will cost £8,000. Towards this the Government has given £3,000, and some £2,000 has been subscribed locally. The hospital treats 800 patients annually, and its accommodation is very limited. At least 32 additional beds are necessary.

The plague mortality in the Punjab is rising by about a thousand a week, and is more than ten times as heavy as it was at this time last year. The following is the official statement of deaths reported from plague during the week ending February 16: Total for the province, 9,237; total for the previous week, 8,110; total for the corresponding week of the previous year, 873.

